# Analysis of the Football Transfer Market Network

**DOI:** 10.1007/s10955-022-02919-1

**Published:** 2022-04-19

**Authors:** Tobias Wand

**Affiliations:** 1 Institut für Theoretische Physik, Westfälische Wilhelms-Universität Münster, Wilhelm-Klemm-Straße 9, 48149 Münster, Germany; 2 CeNoS, Corrensstraße 2, 48149 Münster, Germany

**Keywords:** Complex networks, Football, Soccer, Econophysics, Sociophysics

## Abstract

Using publicly available data from the football database *transfermarkt.co.uk*, it is possible to construct a trade network between football clubs. This work regards the network of the flow of transfer fees between European top league clubs from eight countries between 1992 and 2020 to analyse the network of each year’s transfer market. With the transfer fees as weights, the market can be represented as a weighted network in addition to the classic binary network approach. This opens up the possibility to study various topological quantities of the network, such as the degree and disparity distributions, the small-world property and different clustering measures. This article shows that these quantities stayed rather constant during the almost three decades of transfer market activity, even despite massive changes in the overall market volume.

## Introduction

### Complex Networks

Complex networks can be found in many areas of research, including ecology, sociology, epidemiology, traffic and economics [1, 2]. In particular, financial markets have already been modelled as complex networks, usually by regarding the correlations between different stock time series or their returns as the strength of the edges or using the correlations to construct minimum spanning trees [3]. This approach can uncover clusters between different companies [4] or national indices [5] that can improve portfolio diversification. If the national indices are used as the nodes, regarding the dynamical evolution of a financial network’s synchronisation uncovers pseudo-stationary periods of the market and the correlation between the nodes’ strength and the individual indices’ volatilities helps to classify the national markets into developed, emerging and frontier markets [6]. Similar to the contagion effects for stock markets in [5], power grids can also be considered as dynamical complex networks whose topology strongly affects the stability of the system [7]. Trade networks have already been studied empirically as complex networks [8] and a recent experimental study reveals that the topology of the trade network directly affects the efficiency of the price dynamics [9].

This study aims to apply the methods of network science to the football transfer network as a subclass of trade networks. To the best of the author’s knowledge, this is the first attempt at analysing the football transfer market with the methods of complex network science.

### Football Through the Lens of Socio- and Econophysics

Several sports disciplines have already been analysed through the lens of complex systems [10–12] and football clubs also have already been in the focus of socio- and econophysics research before, with an emphasis on modelling or predicting the outcomes of a match [13] or tournament [14]. An analysis of the stock market shares of football clubs showed only small correlations with the clubs’ home countries’ national indices [15] and inspired by statistical mechanics, a power-law relationship between a team’s win rate and final position leads to the definition of an entropy which allows the assessment of a league’s competitiveness [16]. Network theory has been especially useful for the analysis of football playstyles by modelling each player as a node and a pass as an edge between these nodes [17]. Combining this with real data led to an analysis of different coaches’ strategies [18] and helped to understand the robustness of offensive passing networks against errors and attacks [19]. Related to this network approach is the result in [20]: although coaches tend to change their strategy after a loss, this "Lose-Shift" does not result in an improved success rate because of the co-evolution between antagonistic sides. This adaptation is also assumed to be the reason for the emergence of the power-law behaviour in [16].

### Football Transfer Market

**Fig. 1:**
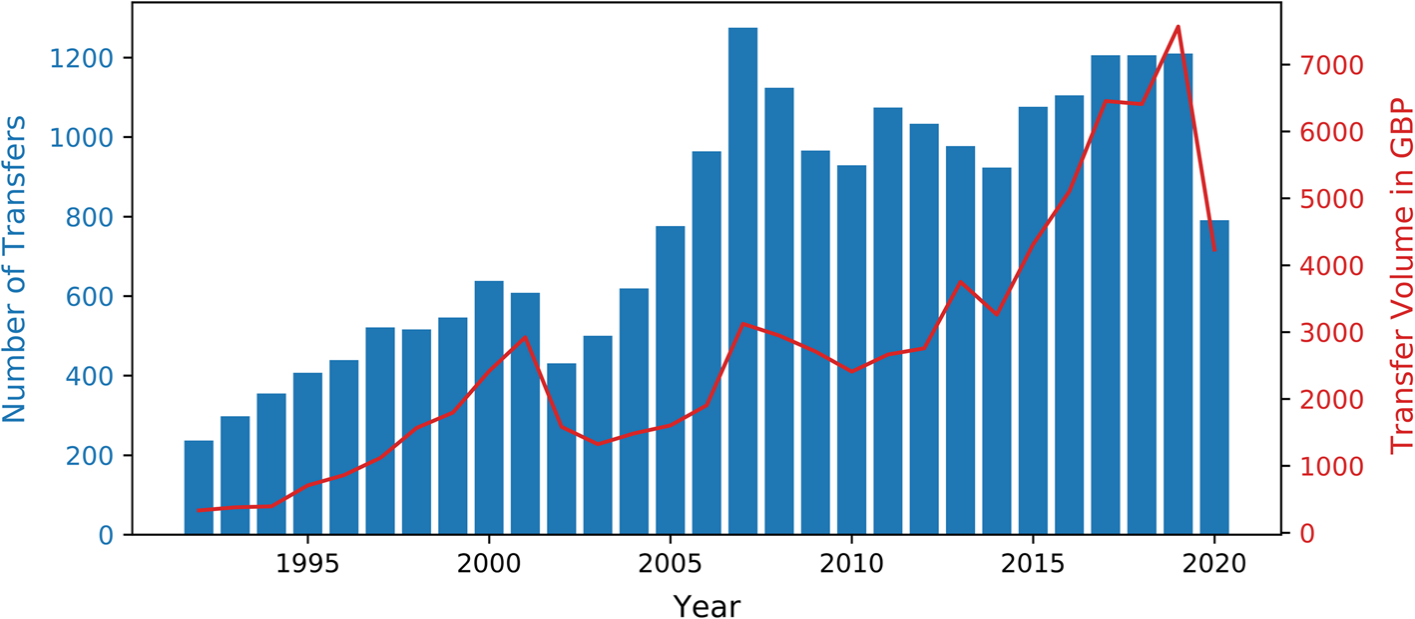
Number of transfers with nonzero transfer fee and total transfer volume in $\pounds$ per year taken from the data in [25]. Like in [24], the 2020 transfer market has clearly shrunk compared to the previous years

European football differs from many American professional sports disciplines by its lack of a drafting system which tries to evenly distribute skilled players among the teams. In European football, players are employees of their clubs and can be transferred to another club before their contract expires, as long as their new club is willing to pay a compensation fee to their current club. Football clubs suffered economically under the pandemic and its accompanying restrictions [21, 22] which lead to a higher risk of bankruptcy among them [23] and an overall decrease of accumulated transfer fees [24]. Figure 1 shows the remarkable decrease in transfer volume in the first year of the pandemic in 2020, but also the general increase in transfer volume in the preceding years. The goal of this study is to find out if the structural properties of the transfer network show any behaviour that is linked to the changes in total transfer volume or whether the market’s structure is independent of the volume.

### Data availability

This article analyses the football transfer market with the methodology of complex networks. The market becomes active during two periods of the season (summer and winter), during which players are transferred from club A to club B for a fee of $X \pounds$. Hence, the transfers of each season form a weighted and directed network. Data on transfers recorded in $\pounds$ is available on [25] and gathered from https://www.transfermarkt.co.uk/ [26] for nine leagues (English Premier League and the corresponding first divisions of German, Spanish, Italian, French, Dutch, Russian and Portuguese football and the second tier English Championship). It records all transfers of all clubs that participated in any of the nine leagues, including those transfers with a club that is not part of any of the top leagues (e.g. an Austrian club). In this paper’s formulation of the network, the source vertex of a transfer is the buyer and the sink is the seller, i.e. the direction of an edge corresponds to the direction of transferred money.

The available data encompasses all years since 1992. Data for the year *Y* includes both the summer transfer window during the summer of year *Y* and the winter transfer window that started in *Y* and ended in $Y+1$. Hence, at the time of writing this article, the data for 2021 is still incomplete due to the ongoing winter 2021/22 transfer period and therefore, 2021 is excluded from this analysis. Many clubs appeared under different names in the data sets (e.g. “1.FC K’lautern” and “1. FC Kaiserslautern”) and therefore had to be matched manually. A rough overview of the total empirical transfer data is given in Fig. 1 and is in line with the findings in [24] that the total transfer volume suffered a steep decline during the Covid Pandemic in 2020, even despite the data sets not being completely identical^1^.

## Methods

### Complex Networks

Following the terminology of [2], a network’s graph *G*(*V*, *E*) is a set of vertices *V* and a set of edges *E* that connect elements of *V*. Mathematically, the edges can be represented by an adjacency matrix *A*. For a binary, undirected graph, that only encodes if there is any connection between two vertices *i* and *j* or not, the adjacency matrix is

1 $$\begin{aligned} A_{i,j} = {\left\{ \begin{array}{ll} 1 &{} \text {if }i\text { and }j\text { are connected} \\ 0 &{} \, \text {else.} \\ \end{array}\right. } \end{aligned}$$

In case of a directed graph, *A* is no longer symmetric and additionally encodes the direction of the edge. For a directed weighted network, the adjacency matrix also contains the strength of the connection from *i* to *j*. Thus, the adjacency matrix generalises to

2 $$\begin{aligned} A_{i,j} = {\left\{ \begin{array}{ll} w_{i,j}&{} \text {if there is a connection from }i\text { to }j\text { with weight }w_{i,j} \\ 0 &{} \, \text {else.} \\ \end{array}\right. } \end{aligned}$$

Naturally, there can also be undirected weighted graphs with a symmetric *A*. This article uses several different representations of the football transfer network because most methods that assess a graph’s structure are only restricted to a certain type of graph. An overview of the different network types and their properties of interest discussed later in this section is given in Table 1.

**Table 1:** Overview of different network representations and their properties

Network type	Property
Binary undirected	Small-world coefficient $\omega$
Binary directed	In- and out-degree power-laws, clustering coefficient from Eq. (4)
Weighted undirected	Disparity power-law
Weighted directed	Clustering coefficient from Eq. (5)

Different types of network representations allow the estimation of different properties. This table provides an overview about which network type is used for the football transfer data to estimate which properties

### Constructing a Network of Football Transfers

For this analysis, the clubs are the vertices and the transfers between them the edges. Only permanent transfers with a nonzero fee are considered as a valid edge between two clubs and disregard e.g. loans, because of two reasons: First, the transferred money between two clubs is used as the weight of an edge between two clubs and therefore, transfers without a fee can be disregarded. And second, including non-permanent transfers can cause the same transfer being accounted for in the transfer networks of multiple years (e.g. Kylian Mbappé was transferred from Monaco to Paris in 2017 on a loan which was transformed into a permanent transfer in 2018). All transfer fees from club *i* to *j* are aggregated as a sum, but notably, there can be transfer streams from *i* to *j* and *j* to *i* simultaneously. For each year, the summer and winter transfer windows are combined because the winter window usually includes only few transfers and therefore may rather be seen as a "correction" of the summer transfer window than as an individual transfer market (cf. [24] for the difference in total transfer fees during summer and winter as an indicator of how small the winter market is compared to the summer market).

### Key Properties of Complex Networks

The following paragraphs explain some of the key properties of complex networks which will be estimated with the football transfer data, namely the degree distribution *p*(*k*), the disparity $Y_2(i)$, the clustering coefficient *C* and the small-world coefficient $\omega$. An overview about which property can be found in which kind of network type is given in Table 1.

A complex network can be characterised by its degree distribution *p*(*k*) which measures the likelihood that a vertex has *k* edges attached to it. For directed networks, this distribution can be split up into in-degree and out-degree distributions. Generally, a power law tail $p(k)\sim k^{-\gamma }$ with $2\le \gamma \le 3$ is associated with a scale-free network [8]. Such a behaviour can be observed if there is a preferential attachment rule during the network’s growth phase [27]. This is to be expected because the transfer network emerges over the course of the entire transfer period (growth) and clubs that have lost a player may seek to replace them by buying another player, therefore having a higher market activity (preferential attachment). Especially relegated or promoted clubs have a necessity to exchange many of their players in order to have a team that is within their current financial restrictions and therefore are expected to have a high number of connections in each direction.

For weighted undirected networks, the disparity $Y_2$ can be used to measure the heterogeneity of the network. For each vertex *i*, the strength $s_i$ of the vertex is defined as the sum of all weights $w_{i,j}$ attached to that vertex [28]. The disparity for each vertex *i* is then defined as

3 $$\begin{aligned} Y_2(i) = \sum _{j \in \nu (i)} \left( \frac{w_{i,j}}{s_i} \right) ^2, \end{aligned}$$

where $\nu (i)$ is the neighbourhood of vertex *i* and contains the $k_i$ vertices connected to *i*. If all attached edges carry similar weight for a non-trivial neighbourhood with $k_i>1$, then $Y_2(i) \sim k_i^{-1}$ and if few weights dominate then $1\ge Y_2>>1/k_i$.

The clustering coefficient has been introduced in [29] and can be interpreted as the probability that two vertices connected to a third vertex are also connected to each other. This quantity has been generalised for weighted and directed complex networks [30]. For binary directed networks, the clustering coefficient of each vertex *i* is

4 $$\begin{aligned} C_i = \frac{(A+A^T)^3_{ii}}{2(d_i^tot (d_i^tot -1)-2d_i^\leftrightarrow )} \end{aligned}$$

with adjacency matrix *A*, its transpose $$A^T$$, $$d_i^tot $$ the total number of connections of the $$i^{th}$$ vertex and $$d_i^\leftrightarrow $$ the number of vertices $$(j_i)_i$$ with a connection to *i* in both directions ($$i\rightarrow j$$ and $$j\rightarrow i$$). Here, the power $$M^\alpha $$ of a matrix *M* means element-wise $$(M_{ij}^\alpha )_{ij}$$. For weighted directed networks, this expression is generalised to

5 $$\begin{aligned} C_i = \frac{\left( A^{1/3}+ (A^T)^{1/3} \right) ^3_{ii}}{2(d_i^tot (d_i^tot -1)-2d_i^\leftrightarrow )}. \end{aligned}$$

Note that for binary networks, $A^{1/3} = A$. In both cases, the overall clustering coefficient for the entire network is the mean of the vertices’ cluster coefficients $C = \sum _{i=1}^N C_i/N$.

Many real-world social systems show a small-world property, i.e. from any vertex *i*, any other vertex *j* can be reached via few edges [29]. Small-world networks are therefore characterised by high clustering coefficients and a low mean path length between two vertices. The quantity $\omega$ defined in [31] measures this property by calculating a trade-off between clustering and shortest path lengths for the analysed network and comparing these quantities to those of idealised networks: Consider a binary undirected network and let *C* and $C_l$ be the clustering coefficients for the given network and for a lattice network, while *L* and $L_r$ are the mean shortest distance between any two nodes of the given network and of a random network, then $\omega$ is defined as

6 $$\begin{aligned} \omega = L_r/L - C/C_l. \end{aligned}$$

$\omega$ is restricted to $[-1,1]$ and places the network on a continuum from regular lattices ($\omega \approx -1$) to random graphs ($\omega \approx 1$) and is more robust than the similar small-world measure $\sigma$ from [29] given as

7 $$\begin{aligned} \sigma = \frac{C}{C_r} / \frac{L}{L_r} \end{aligned}$$

with $C_r$ being the clustering coefficient of a random graph. The computation of the clustering coefficients and of the small-world $\omega$ is done via the package NetworkX in python [32] with the functions *networkx.algorithms.cluster.clustering* and *networkx.algorithms.smallworld.omega*.

## Results

### Degree Distributions

**Fig. 2:**
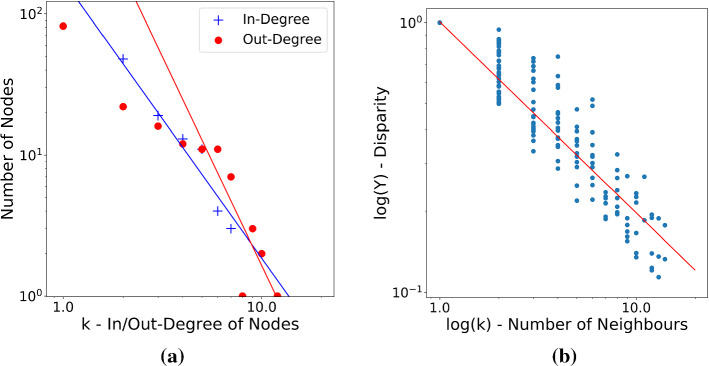
Exemplary fit of the power law for the 1997 data and **a** the in-degree and out-degree distribution with the linear fits at their tails and **b** the disparity *Y*

If the annual football transfer networks are simplified to a binary directed network, then their in-degree distributions follow a power law as seen in the sample Fig. 2a. The out-degree distributions roughly display a power law behaviour for $k_{out}\ge 5$ and a different power law scaling for smaller *k*. The following reasoning might account for the difference between the in- and out-degree distributions: Many clubs outside of the top 9 leagues are contained in the data set because some of their players have been bought by one of the clubs from the top leagues. But only those transfers are recorded where at least one participant (buyer/seller) is a member of the top 9 leagues. Therefore, many transfers between clubs from smaller leagues are not included in our data because both participants are from smaller leagues. Hence, several clubs from smaller leagues with only one or two out-degree edges (i.e. bought players) actually would have more out-degree edges if even smaller leagues were included in the data, too. Thus, the difference between in- and out-degree distributions seems to reflect the incompleteness of the recorded data.

The exponent $\gamma _{in}$ of the in-degree distribution $p_{in}(k)\sim k^{-\gamma _{in}}$ is estimated via linear regression to the log-log-scaled data and lies between 1.6 and 2.4 for each year. For all annual networks, the regressions’ coefficients of determination $R^2$ values are above 0.88 which indicates a good fit. For all but one year (2019), the range of scale-free exponents $2\le \gamma \le 3$ overlaps with the $2\sigma$ confidence interval of $\gamma _{in}$.

### Disparity Distribution

**Fig. 3:**
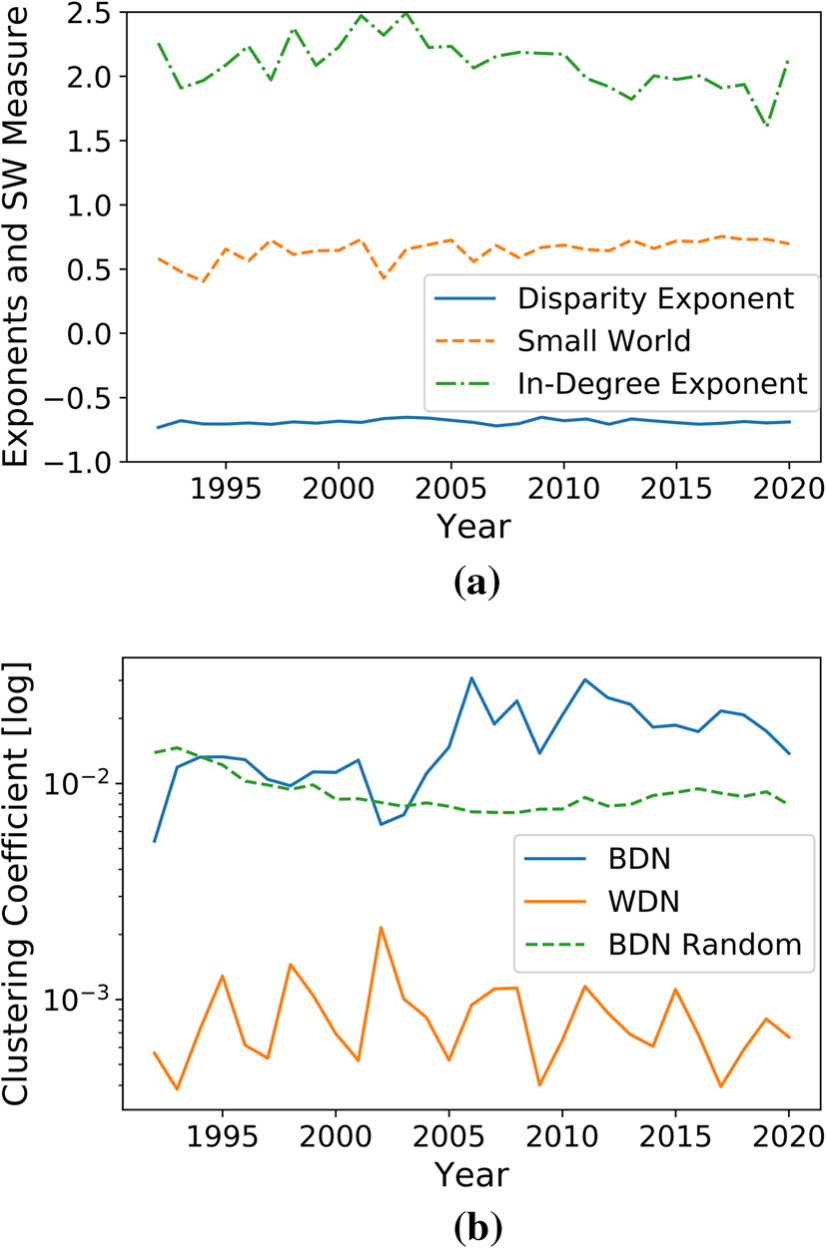
**a** The small-world coefficient $\omega$ and the exponents $\gamma _{Y_2}$ of the disparity distribution $Y_2$ and $\gamma _{in}$ of the in-degree distribution $p_{in}$. **b** Clustering coefficients for the transfer network as a binary directed network (BDN) and weighted directed network (WDN) compared to the clustering coefficient of a random binary directed network with the same average number of edges per vertex

If the data is treated as a weighted undirected network (i.e. transfers from $i\leftarrow j$ and $j \leftarrow i$ are combined to one transfer stream $i\leftrightarrow j$), the disparity distribution shows a clear power-law behaviour as illustrated in Fig. 2b. The power-law exponent $\gamma _{Y_2}$ of the disparity is rather constant over time as shown in Fig. 3a and varies between 0.65 and 0.73, meaning that the disparity follows a power law $Y_2(i) \sim k_i^{-\gamma _{Y_2}}$ with exponent $\gamma _{Y_2}$ close to one. This suggests that for each vertex, the edges attached to it have rather heterogeneous weights. All fits show a coefficient of determination $R^2>0.85$, meaning that the power law fit is highly trustworthy.

### Clustering and Small-World

**Fig. 4:**
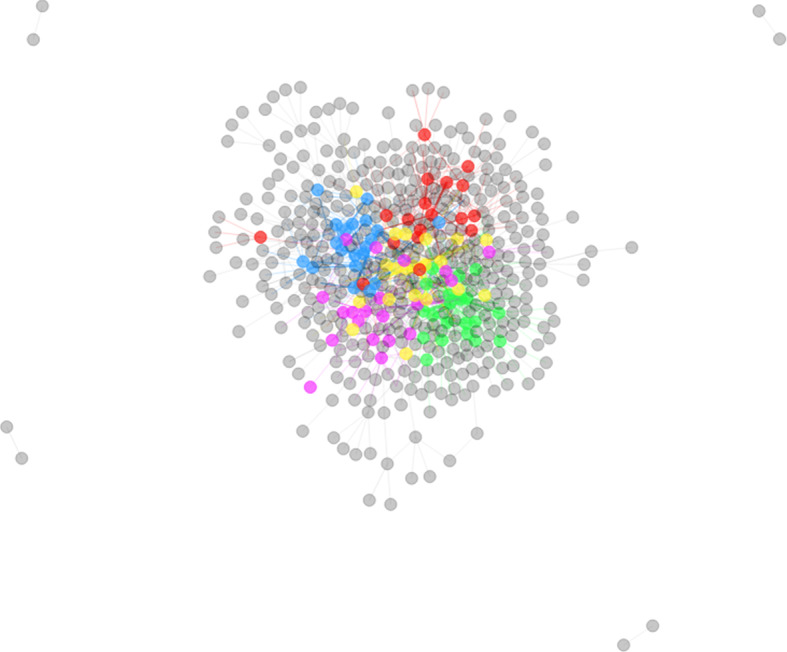
Visualisation of all transfers in 2011 for the top 9 leagues. Red points denote clubs from the German Bundesliga, blue the English Premier League, green the Italian Serie A, yellow the Spanish Primera División and violet the French Ligue 1. Thickness of the edges represents the amount of transfer fees, but is difficult to discern on this image. Almost all clubs are connected to the giant component. This graphic was created with the python package pyvis [33]

The clustering coefficients for the binary directed network (simply asking if there was any transfer from *i* to *j*) and for the weighted directed network are depicted in Fig. 3b. Also, clustering coefficients of a random binary directed network with the same average number of edges per vertex is shown as a comparison. Interestingly, the BDN version of the network shows a rather good correspondence to that or a random BDN until 2003, but always exceeds the random BDN’s coefficient afterwards, indicating that there is a small tendency towards clusters of three interconnected clubs only after 2003. Potentially, this may reflect geographical clusters (e.g. only English clubs) or at the top level of competition clusters between e.g. Champions League participants. Figure 4 (made with the python package pyvis [33]) in the appendix indicates the existence of geographic clusters for the year 2011. The WDN network always shows a lower clustering coefficient than the BDN which is in line with the rough theoretical estimation in Eq. 11 of [30] that for weights uniformly distributed on [0, 1], the cluster coefficients fulfil the inequality $C_{WDN} < C_{BDN}$.

To estimate the small-world measure $\omega$ with the function *networkx.algorithms.smallworld.omega* from the package [32], one has to restrict the data to the fully connected giant component of the network which encapsulates more than 90% of the data as demonstrated in Fig. 4 in the appendix with an example. This is in line with the procedure of e.g. [29] (cf. their description of Table 1). It should be noted that older networks tend to have more vertices disconnected from the giant component. $niter=20$ rewirings per edge and $nrand=10$ generated graphs are used for the estimation of $\omega$ with a constant random seed for all years. The small-world measure $\omega$ is always in the interval (0.40, 0.76) and depicted in Fig. 3a. Telesford et al. admit that a clear definition of a small-world range for $\omega$ is difficult, but advocate for $-0.5\le \omega \le 0.5$ being an indicator of a small-world network [31]. The estimated $\omega$ only falls within this interval for the networks of 1993, 1994 and 2002 and therefore does not provide clear evidence towards a small-world behaviour.

## Conclusion and Outlook

### Summary

This research article shows on the one hand that the football transfer network displays classical properties of complex networks (the power law distributions), but on the other hand, it cannot verify the small-world characteristic. Because of the ubiquity of small-world networks, the non-small-world characteristic is unexpected, but may indicate that clubs restrict their transfer activities to e.g. their domestic competitors and/or to clubs in the same competitive weight class (e.g. other UEFA Champions League participants). The disparity exponent of $\gamma _{Y_2}\sim 0.7$ indicates homogeneity among the weights of any vertex’s edges, meaning that clubs usually do not have a transfer that is outstandingly more expensive than their other transfers, which further indicates that clubs restrict their transfer activities to other clubs with similar financial resources. There is an asymmetry between the in-node and out-node distributions, but this probably has to be attributed to the available data.

Interestingly, the binary clustering coefficient is close to the coefficient of a random network before 2003, but displays slightly higher clustering afterwards. However, the other graphs in Fig. 3 do not show any clear time-dependent trend. In particular, the Covid-19-struck 2020 transfer window shows no obviously different behaviour than the previous years: Although the transfer market has shrunk because of Covid-19 [24], this analysis did not reveal a change in the market’s network structure. This might indicate that all clubs were affected similarly by the economic effects of the pandemic irrespecitve of e.g. the club’s size or revenue. In contrast, the network analysis of global financial markets showed that the financial market network experienced numerous differences in its topological structure and revealed evidence for a contagion effect among lesser developed markets [5].

### Future Research and Implications for Regulators

Further insight into the transfer network could be gained by increasing the amount of data, especially with respect to lower-level leagues as only the English second league has been included in the data set. In particular, it would be interesting to see if lower-level clubs show a higher degree of clustering because of the less international scope of their transfer markets. However, one might have to deal with less trustworthy and accurate data, especially with regards to the exact transfer fees. Additionally, many lower-level clubs barely pay any fees for their new players because of a lack of financial resources. If one wishes to include more lower-level clubs into the analysis, it might therefore make sense to evaluate all transfers instead of only those with a nonzero fee. Then, the amount of transferred players between two clubs could be used as the weight of their edge instead of the combined fees. Such a network model would also open up the possibility of a multi-layered network [34] and treat e.g. clubs from the first or second league as different layers of the transfer network.

Also, one can devise a multi-layered network which devotes one layer to the individual clubs and one layer to the different countries. On the level of different countries, one can define the proximity between two countries by the relative share of transfers between their leagues versus transfers with other countries similar to the correlation between time series in [6] to follow that publication’s approach to detecting synchronisation in the transfer network.

Although the football transfer network seems to have a higher topological resilience against the impact of Covid than e.g. the stock index network, there is nonetheless the threat of experiencing errors in the network [35] or cascading failures known from other empirical networks [36–38]. Regulators are recommended to focus on the possibility of cascading failures or contagion like in [5] for the football transfer network and to estimate the effect of e.g. one club going bankrupt and disrupting the flow of money through the market network similar to the Lehman Brothers bankruptcy that started the financial crisis in 2008. For this purpose, it may be worth to investigate the effects that legal obstacles to player transfers (e.g. the minimum quota of homegrown players in England or the limit to non-EU players in Spain) have on the stability of the market network. Because [9] pointed out the inefficiency of a price process in small-world networks compared to random ones, regulators should also be interested in preventing the football transfer market from adopting the small-world topology.

## Appendix A: Visualisation of the Network

As an example, the transfers in 2011 are shown with colour coding for the five major leagues: Only the clubs from the “big-five leagues” in [24] have been coloured and their proximity indicates the existence an English (blue) and Italian (green) local cluster.

## Footnotes

1 The analysis in [24] only considers the English, Spanish, Italian, German and French first tier leagues.
